# Explaining income-related inequalities in cardiovascular risk factors in Tunisian adults during the last decade: comparison of sensitivity analysis of logistic regression and Wagstaff decomposition analysis

**DOI:** 10.1186/s12939-019-1047-6

**Published:** 2019-11-15

**Authors:** Olfa Saidi, Nada Zoghlami, Kathleen E. Bennett, Paola Andrea Mosquera, Dhafer Malouche, Simon Capewell, Habiba Ben Romdhane, Martin O’Flaherty

**Affiliations:** 1 Cardiovascular Epidemiology and Prevention Research Laboratory –Faculty of medicine of Tunis, University Tunis El Manar, Tunis, Tunisia; 2 National Institute of Health, Tunis, Tunisia; 3 Population and Health Sciences, Royal College of Surgeons in Ireland, Dublin, Ireland; 4 Department of Epidemiology and Global Health, Umeå University, Umeå, Sweden; 5 National Institute of Statistics and Data Analysis Tunis, Tunis, Tunisia; 6 Department of Public Health and Policy, University of Liverpool, Liverpool, UK

**Keywords:** Social inequalities, Global sensitivity analysis (GSA), Logistic regression, Wagstaff-type decomposition analysis, Diabetes, Cardiovascular risk factors, Tunisia

## Abstract

**Background:**

It is important to quantify inequality, explain the contribution of underlying social determinants and to provide evidence to guide health policy. The aim of the study is to explain the income-related inequalities in cardiovascular risk factors in the last decade among Tunisian adults aged between 35 and 70 years old.

**Methods:**

We performed the analysis by applying two approaches and compared the results provided by the two methods. The methods were global sensitivity analysis (GSA) using logistic regression models and the Wagstaff decomposition analysis.

**Results:**

Results provided by the two methods found a higher risk of cardiovascular diseases and diabetes in those with high socio-economic status in 2005. Similar results were observed in 2016.

In 2016, the GSA showed that education level occupied the first place on the explanatory list of factors explaining 36.1% of the adult social inequality in high cardiovascular risk, followed by the area of residence (26.2%) and income (15.1%). Based on the Wagstaff decomposition analysis, the area of residence occupied the first place and explained 40.3% followed by income and education level explaining 19.2 and 14.0% respectively. Thus, both methods found similar factors explaining inequalities (income, educational level and regional conditions) but with different rankings of importance.

**Conclusions:**

The present study showed substantial income-related inequalities in cardiovascular risk factors and diabetes in Tunisia and provided explanations for this. Results based on two different methods similarly showed that structural disparities on income, educational level and regional conditions should be addressed in order to reduce inequalities.

## Key messages

- Our study explains the income-related inequalities in cardiovascular risk factors among Tunisian adults during the last decade.
- It compares two methods (sensitivity analysis of logistic regression model and Wagstaff decomposition analysis).
- Both methods pointed tofound similar factors explaineding inequalities (income, educational level and regional conditions (area and region)) but with different rankings of importance.

## Introduction

Inequalities in health are considered unacceptable as Alma-Ata declared in 1978 by stating that “the existing gross inequality in the health status of the people particularly between developed and developing countries as well as within countries is politically, socially and economically unacceptable and is, therefore, of common concern to all countries” [1]. Accordingly, the World Health Organization (WHO) has issued a call to action to reduce health inequalities [2].

Several epidemiological studies have examined the income-related inequalities of noncommunicable diseases, in particular cardiovascular diseases (CVDs) and diabetes. Cardiovascular risk factors are well documented in Tunisia [3, 4]. However, such studies have been unable to address policy makers concerns on which interventions and preventive strategies will be most effective in reducing the observed inequalities and how to address the groups most in need of intervention [5, 6].

The major limitation of Tunisian and international research on income-related inequalities of cardiovascular diseases and diabetes is the dominant focus on the existence of inequalities without identifying their basis, which would allow greater understanding of the reasons for inequalities [7–10]. Some advanced statistical methods can help overcome many of these limitations playing a crucial role in helping to quantify inequality and explain the contribution of the underlying social determinants as well as to guide health policies and the development of strategies at different levels [11, 12].

In this context, this study aimed to quantify the income-related inequalities in five cardiovascular risk factors (diabetes, hypertension, obesity, hypercholesterolemia, smoking and high cardiovascular risk) in the last decade in Tunisia, and to determine the contribution of a broad range of social determinants of the inequalities. In order to identify the most explanatory social determinants, we used the two main approaches currently used to explain social inequalities, a global sensitivity analysis using logistic regression model and the Wagstaff-type decomposition analysis.

## Methods

### Study population

To study the evolution of income-related inequalities of cardiovascular risk factors in Tunisia during the last decade, we used two large national surveys: (i) the Epidemiological Transition and Health Impact in North Africa Survey in 2005 (TAHINA-2005) including 8007 individuals aged 35–70 years old (3417 men and 4590 women) and (ii) the Tunisian Health Examination Survey (THES-2016) in 2016 including 6007 individuals aged 35–70 years old (2859 men and 3148 women).

The two surveys used were representative of the underlying population and were based on the same sampling method. The definitions of cardiovascular risk were the same except for diabetes in 2016 where the Hemoglobin A1C was integrated as recommended by the American Diabetes Association.

Details of the study population were presented in the technical appendix.

### Measurements

#### Socio-economic and demographic variables

The variables used as social determinants in our study included demographic and socio-economic factors that had plausible links with cardiovascular risk factors and diabetes [13, 14].

Demographic factors included four variables: (i) gender defined as male coded “1” and female coded “2”; (ii) age categorized into four groups: “1” 35–44, “2” 44–54, “3” 55–64 and “4” 65–70 years; (iii) geographic area (“1” Urban, “2” Rural) and (iv) region of residence categorized into7 administrative regions of the country (“1” District of Tunis, “2” North-East, “3” North-West, “4” Central-East, “5” Central-West, “6” South-East and South-West).

Data on geographic area and region of residence was obtained from the sampling frame derived by the Tunisian National Institute of Statistics [15, 16]. Age and gender were self-reported by the survey participants.

Socio-economic factors included level of education, professional occupation and quintile of well-being. Data were self-reported by the survey participants.

(i) Education was classified according to the classification of National Tunisian Institute of Statistics into four categories [17]: level 0, Never educated “illiterate”; level 1, low level education “Primary” (6 years of schooling); level 2, intermediate-level education “Secondary” (7–13 years of schooling) and level 3, higher education “university and higher (> = 14 years of schooling).
(ii) The classification of the professional activity status is defined according to the structure of the International Standard Classification of Occupations [18] and categorized into four groups: 1 higher level Senior level; 2 Intermediate level; 3 workers and 4 unemployed or retirees.
(iii) Quintile of income: income of household as categorized into quintiles with quintile 1 representing the lowest income and quintile 5 representing the highest.

#### Cardiovascular risk factors

##### Hypertension

In both surveys (TAHINA-2005 and THES-2016), three blood pressure readings were measured separately; the mean systolic blood pressure (SBP) and diastolic blood pressure (DBP) of the three measures were considered. Based on WHO recommendations the following is considered hypertensive; any person with a SBP > = 140 and / or DBP > = 90 mmHg and / or who claims to be diagnosed for arterial hypertension and / or treated [19].

##### Diabetes

For TAHINA Survey in 2005, diabetes prevalence was defined using the WHO criteria [20] as Fasting Plasma Glucose (FPG) ≥ 7 mmol/L, confirmed medication usage from the medication inventory, self-reported use of antidiabetic medications within the past 2 weeks of the examination, or self-reported diabetes diagnosis.

For THES Survey in 2016, diabetes prevalence was defined according to the definition of the American Diabetes Association (ADA) as FPG > =7 mmol/L or postprandial glucose > = 11 mmol/L, Hemoglobin A1C (HbA1C) > = 6.5%, self-reported diabetes diagnosis or confirmed medication usage from the medication inventory [21, 22].

##### Hypercholesterolemia

In both surveys (TAHINA-2005 and THES-2016), the prevalence of hypercholesterolemia is defined by total serum cholesterol ≥6.2 mmol /l or triglyceride ≥3 mmol / or self-reported hypercholesterolemia diagnosis [23].

##### Obesity

In both surveys (TAHINA-2005 and THES-2016), obesity is defined by Body Mass Index (BMI) > = 30 Kg / m^2^ [24].

##### Smoking

In both surveys (TAHINA-2005 and THES-2016), the prevalence of smoking is defined by the daily consumption of tobacco at the time of the survey.

##### High cardiovascular risk

In both surveys (TAHINA-2005 and THES-2016), high cardiovascular risk is defined by the cumulative number of risk factors ≥3, out of the five mentioned above.

In the main manuscript, we only present results of the high cardiovascular risk. The analysis of other individual factors (Diabetes, Tobacco, obesity, hypertension and hypercholesterolemia) are presented in the technical appendix.

### Statistical analysis

#### Sensitivity analysis to select the most influencing risk factors

The Global Sensitivity Analysis (GSA) was defined as how the uncertainty in the output of a model can be apportioned to the different sources of uncertainty in the model input. The method quantifies the contribution of uncertainty in different social determinants (inputs) to a specific output variable of interest (the disease) [25, 26].

The GSA in this study is generally based on the sensitivity index for measuring the importance of a given social determinant on the output variable designating the disease (sick, not sick, eg hypertension).

The sensitivity index of a determinant is defined by the fractional contribution to the variance of the output variable.

#### GSA in a logistic regression model

In this study, we are interested in the sensitivity analysis of the logistic regression model.

The theory of constructing a logistic regression model is detailed in the appendix [27, 28].

The process of selecting the important covariates from the available set of covariates and constructing an appropriate logistic regression model involved three steps:

1. To identify the probability distribution f (x_i_) of each covariate in the model.
2. The logistic regression model, in terms of Logit (as in equation below) and the information about the covariates obtained in step one are used to create a Monte Carlo simulation to generate the sample that will be used in the decomposition and to estimate the unconditional variance of the response probability and the conditional variance for covariates.
3. The results from step two will be used in performing GSA in the binary logistic regression model and in the decomposition analysis, resulting in the estimate of S_i_. In this step, we refer to the Sabol method [28] using R software.

#### Wagstaff-type decomposition analysis

The decomposition analysis quantifies the degree of income-related inequalities and explains the contribution of each factor to the observed inequality. The decomposition approach is the analysis of the contribution that each of the inequalities in the social determinants has on the inequalities in the observed disease.

This analysis is based on the concentration curve (CC) and the concentration index (C) [29].

The analysis was done through the following steps:

1. Run a regression analysis;
2. Compute the elasticity (weighted coefficient);
3. Calculate the concentration indexes of the covariates;
4. Calculate the contributions.

Details of the method were presented in the technical appendix.

## Results

### Socioeconomic and demographic characteristics of the study populations

Table 1 shows the socioeconomic and demographic characteristics of the study populations.

**Table 1:** Socioeconomic and demographic characteristics of the population studies in 2005 and 2016

Study	TAHINA-2005 *N* = 7553		THES-2016 *N* = 5449	
	N	Weighted %	N	Weighted %
Characteristics				
Gender				
Male	3233	49.6	2610	49.8
Female	4320	50.4	2839	50.2
Age-Groups (years)				
35–44	2992	43.2	1803	37.5
45–54	2469	31.0	1708	31.5
55–64	1319	17.2	1443	23.4
65–70	773	8.5	495	7.6
Mean age	49.0 ± 9.5		49.2 ± 9.7	
Area				
Urban	4372	67.7	3606	67.6
Rural	3181	32.3	1843	32.4
Region				
District of Tunis	925	24.4	685	23.2
North East	1024	14.0	799	15.0
North West	1124	13.0	886	11.8
Centre East	1099	22.4	836	24.6
Centre West	1176	12.3	763	11.9
South East	1070	8.4	708	8.0
South West	1135	5.5	772	5.5
Level of education				
Illiterate	3258	34.9	1240	19.3
Primary	2588	35.1	2117	39.2
Secondary	1233	20.1	1472	28.9
University	474	9.8	620	12.6
Occupation				
Upper	980	15.8	484	10
Intermediate	359	6.0	317	6.8
Employee/worker	2370	35.0	2187	43.4
Unemployed/retired	3844	43.2	2461	39.9
Household income				
1st quintile	1510	15.7	1089	18.1
2nd quintile	1510	17.3	1090	19.0
3rd quintile	1511	20.6	1090	20.9
4th quintile	1511	21.0	1090	21.5
5th quintile	1511	25.4	1090	20.5

In 2005, the mean age of the study population was 49.0 ± 9.5 years. Women accounted 50.4% of the study population and 68.0% lived in an urban area of residence. About one in four people were residing in the District of Tunis. 34.9% were illiterate, as well 43.2% were unemployed or retired. Additionally, 15.7% of the surveyed population lived at the lowest quintile in 2005.

In 2016, the mean age was 49.2 ± 9.7 years, and women accounted for 50.2% of the study population. The percentage of urban population was 67.6%. In addition, 23.2% of the study population were residents in the District of Tunis region and 19.3% reported having no education in 2016. In addition, 39.9% were unemployed or retired. The percentage of the surveyed population lived at the lowest quintile was 18.1%.

### High cardiovascular risk in the last decade by social determinants

#### Evolution of high cardiovascular risk prevalence in the last decade by social determinants

Table 2 presented the high cardiovascular risk prevalence by social determinants in 2005 and 2016. In 2005, 12.4% [11.4–13.3] of the adults aged 35–70 years old had high cardiovascular risk (13.6% [12.0–15.0] among men and 11.2% [10.1–12.4] among women).

**Table 2:** Evolution of the prevalence of high cardiovascular risk prevalence by social determinants between 2005 and 2016

Study	TAHINA-2005		THES-2016	
	N	High cardiovascular risk prevalence %[CI 95]	N	High cardiovascular risk prevalence %[CI 95]
Characteristics				
Gender		***P*** **= 0.110**		***P*** **= 0.269**
Male	3233	13.6 [12.0–15.0]	2610	21.1 [19.2–22.9]
Female	4320	11.2 [10.1–12.4]	2839	19.6 [17.9–21.3]
Age-Groups (years)		***p*** **< 10**^**−3**^		***P*** **< 10**^**−3**^
35–44	2992	7.4 [6.2–8.7]	1803	11.2 [9.4–13.0]
45–54	2469	14.1 [12.3–15.9]	1708	22.9 [20.6–25.3]
55–64	1319	19.1 [16.5–21.7]	1443	27.7 [25.0–30.3]
65–70	773	17.7 [14.5–21.0]	495	31.9 [27.3–36.4]
Area		***P*** **< 10**^**−3**^		***P*** **< 10**^**−3**^
Urban	4372	14.8 [13.5–16.1]	3606	22.8 [21.2–24.4]
Rural	3181	7.3 [6.3–8.3]	1843	15.1 [13.3–17.0]
Region		***P*** **< 10**^**−3**^		***p*** **= 0.003**
District of Tunis	925	19.2 [16.3–22.0]	685	22.4 [19.1–25.7]
North East	1024	9.5 [7.6–11.4]	799	20.2 [17.3–23.0]
North West	1124	6.6 [5.2–8.1]	886	19.0 [16.3–21.7]
Centre East	1099	15.1 [12.9–17.2]	836	22.1 [19.6–25.3]
Centre West	1176	7.9 [6.3–9.4]	763	16.2 [13.5–18.8]
South East	1070	8.8 [6.9–10.6]	708	16.2 [13.4–19.0]
South West	1135	8.0 [6.4–9.6]	772	20.3 [17.4–23.3]
Level of education		***P*** **= 0.057**		***P*** **= 0.003**
Illiterate	3258	10.7 [9.4–11.9]	1240	22.9 [20.2–25.6]
Primary	2588	12.3 [10.7–13.9]	2117	21.1 [19.1–23.1]
Secondary	1233	15.9 [13.3–18.4]	1472	19.6 [17.3–22.0]
University	474	11.7 [8.3–15.1]	620	15.6 [12.1–19.0]
Occupation		***P*** **= 0.021**		***P*** **= 0.038**
Unemployed/retired	3844	11.5 [10.2–12.8]	2461	22.6 [20.7–24.5]
Employee/worker	2370	12.2 [10.6–13.9]	2187	18.9 [17.0–20.8]
Intermediate	359	14.9 [10.4–19.4]	317	18.9 [13.7–24.1]
Upper	980	14.2 [11.5–16.9]	484	18.6 [14.4–22.8]
Quintile income		***P*** **< 10**^**−3**^		***P*** **= 0.017**
1st quintile	1510	6.9 [5.4–8.3]	1089	18.2 [15.6–20.8]
2nd quintile	1510	9.0 [7.2–10.7]	1090	18.7 [16.0–21.4]
3rd quintile	1511	12.2 [10.2–14.3]	1090	21.9 [19.1–24.7]
4th quintile	1511	14.9 [12.6–17.1]	1090	22.5 [19.6–25.4]
5th quintile	1511	16.2 [13.9–18.5]	1090	19.8 [17.0–22.6]
Total	**7553**	**12.4 [11.4–13.3]**	**5449**	**20.3 [19.1–21.6]**

*CI 95* 95% Confidence Interval

p: *p* value at 5% significance level

A greater prevalence of high cardiovascular risk was observed in those aged 55–64 years of age (19.1% [16.5–21.7]) compared to those aged between 35 and 44 years (7.4% [6.2–8.7]). High cardiovascular risk was twice as high in the urban area (14.8% [13.5–16.1]) compared to the rural area (7.3% [6.3–8.3]) and three times higher in the District of Tunis (19.2% [16.3–22.0]) than in the north-west (6.6% [5.2–8.1]). The High cardiovascular risk prevalence was more dominant among people with secondary education level (15.9% [13.3–18.4]) than other educational levels and among those intermediate occupation level (14.9% [10.4–19.4]).

The highest prevalence was observed among people within the highest income quintile (16.2% [13.9–18.5]) compared to the lowest quintile (6.9% [5.4–8.3]).

In 2016, the high cardiovascular risk prevalence increased to reach 20.3% [19.1–21.6] among Tunisian adults aged between 35 and 70 years: 21.1% [19.2–22.9] among men and 19.6% [17.9–21.3] among women. This prevalence increased gradually with age in 2016 where it was 11.2% [9.4–13.0] in those aged 35–44 years reaching 31.9% [27.3–36.4] in those aged 65–70 years of age.

In 2016, the highest prevalence of high cardiovascular risk was observed in the urban area (22.8% [21.2–24.4]) and in the district of Tunis (22.4% [19.1–25.7]). Additionally, the prevalence of high cardiovascular risk was particularly high among illiterate participants (22.9% [20.2–25.6]), the unemployed and retired (22.6% [20.7–24.5]) and within the fourth income quintile (22.5% [19.6–25.4]) (Table 2).

#### Global sensitivity analysis in a logistic regression model method of high cardiovascular risk

The global sensitivity analysis in a logistic regression model, reflecting the ranking of variables ordered by the level of importance and contribution of each o explaining the total variance of the high cardiovascular risk prevalence, showed that the most influential factors were the geographical area, the region of residence and the income contributing 30.7, 21.4 and 20.1% respectively in 2005.

In 2016, the education level occupied the first place (36.1%), followed by the area (26.2%) and the income (15.1%). The occupation level represented 11.7% of the total variance (Table 3).

**Table 3:** Sensitivity indices and social determinants ranking of high cardiovascular risk

Social determinants	2005			2016		
	S_I_	S_i_ (%)	Rank	S_I_	S_i_ (%)	Rank
Gender	0.009	0.9	7	0.006	0.4	7
Age	0.117	11.1	5	0.031	2.3	6
Area	0.323	30.7	1	0.35	26.2	2
Region	0.225	21.4	2	0.109	8.1	5
Education level	0.045	4.3	6	0.483	36.1	1
Occupation	0.122	11.6	4	0.157	11.7	4
Household income	0.212	20.1	3	0.202	15.1	3
Total	**1.053**	**100.0**		**1.338**	**100.0**	

*S*_*I*_ Sensitivity indice

#### Wagstaff-type decomposition analysis of high cardiovascular risk prevalence

##### Income-related inequality in high cardiovascular risk prevalence

**Fig. 1:**
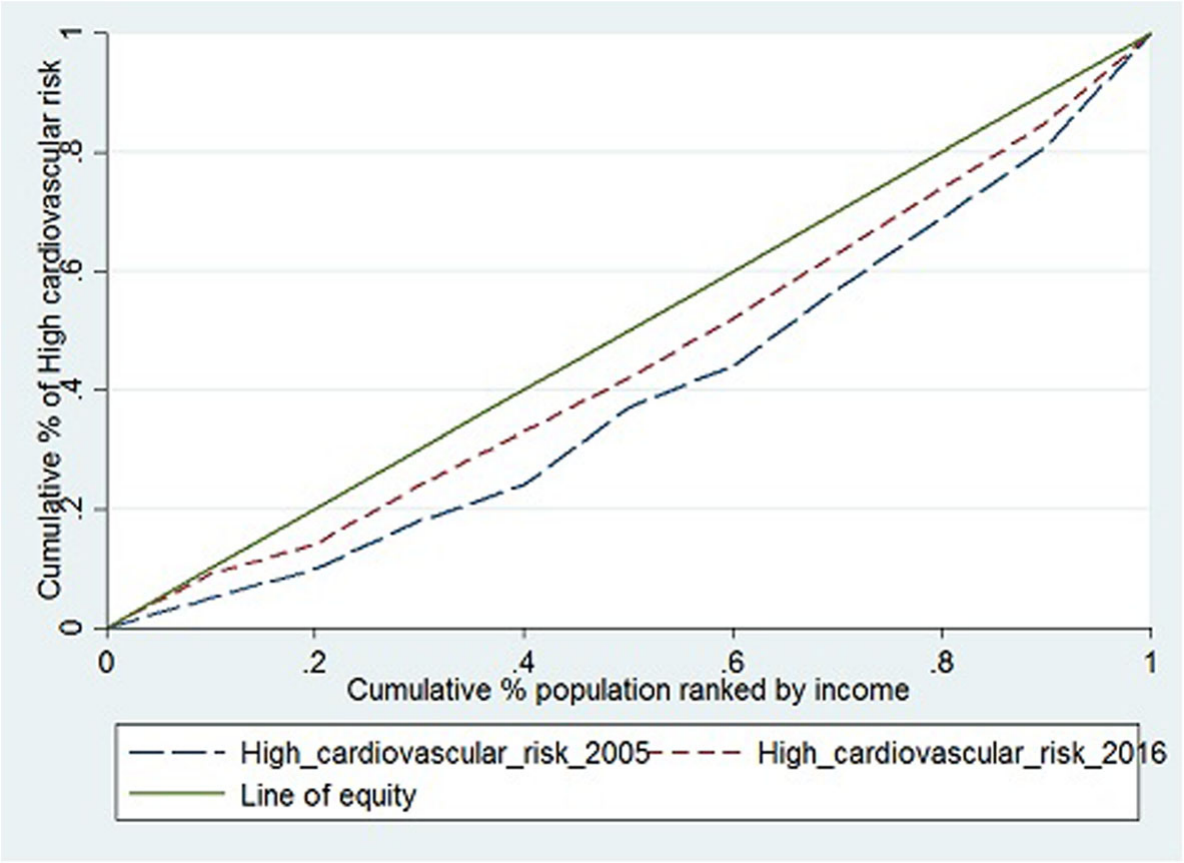
The concentration curve of income-related inequality in high cardiovascular risk among Tunisian adults in 2005 and 2016

The concentration curves of income-related inequalities of high cardiovascular risk prevalence in 2005 and 2016 are presented in Fig. 5. The concentration curve lies under the diagonal line of equity, with the overall concentration index is estimated at 0.116 (95% CI: 0.092 to 0.140) in 2005 and 0.085 (95% CI 0.048 to 0.122) in 2016. High cardiovascular risk was mainly concentrated among those with the highest income in both observation periods (Fig. 1).

##### Decomposition analysis of high cardiovascular risk

The Wagstaff decomposition analysis of high cardiovascular risk found that, in 2005, regional conditions had a principal role in explaining the income-related inequalities in high cardiovascular risk: The geographical area was in first place (34.6%) followed by the region (21.8%). The education level and occupation were ranked next explaining 13.8 and 13.2% respectively. Then, income explained 10.8%. Lastly, age and gender contributed 4.8 and 1.0% to the observed inequalities respectively.

In 2016, the geographical area still occupied the first place and explained 40.3% of the adult social inequality in high cardiovascular risk followed by the income, education level and occupation with contributions of 19.2, 14.0 and 13.6% respectively. The age and the region represented 6.3 and 4.6% of the observed inequalities respectively. Gender only explained 2.1%.

Based on the positive concentration indices in 2005 and 2016, men, people aged 45 to 54 years, residents in urban areas, residents in the North-East and Central-East, those with secondary education, middle managers and those belonging to the highest income quintiles were mainly concentrated in the richer population. These groups contributed to the inequalities in the prevalence of high cardiovascular risk.

Furthermore, the social determinants included in the model accounted for 53 and 51% of the estimated inequality of high cardiovascular risk in Tunisia respectively in 2005 and 2016, based on the residual, reflecting the unexplained part of the inequality in terms of prevalence of high cardiovascular risk (Table 4).

**Table 4:** Decomposition of the concentration index of high cardiovascular risk prevalence among Tunisian adults in2005 and 2016

	2005						2016					
	Coef	Elast	CI	Cont to C	% cont	% Adj	Coef	Elast	CI	Cont to C	% cont	% Adj
Gender						**1.0**						**2.1**
Female (Ref)												
Male	0.004	0.015	0.129	0.002	1.7	1.0	0.021	0.048	0.070	0.003	3.9	2.1
Age						**4.8**						**6.3**
35–44 years (Ref)						0.0						0.0
45–54 years	0.071	0.221	0.043	0.010	8.2	4.8	0.133	0.203	0.050	0.010	12.0	6.3
55–64 years	0.150	0.249	− 0.032	− 0.008	−6.9	0.0	0.184	0.238	−0.081	− 0.019	− 22.7	0.0
65–70 years	0.162	0.158	− 0.157	− 0.025	− 21.3	0.0	0.238	0.106	− 0.179	− 0.019	− 22.2	0.0
Area						**34.6**						**40.3**
Rural (ref)						0.0						0.0
Urbain	0.032	0.174	0.398	0.069	59.5	34.6	−0.079	−0.131	− 0.497	0.065	76.4	40.3
Region						**21.8**						**4.6**
District of Tunis (Ref)						0.0						0.0
North_east	−0.049	−0.063	0.014	−0.001	− 0.7	0.0	0.006	0.004	0.043	0.000	0.2	0.1
North_west	−0.060	− 0.085	−0.232	0.020	17.0	9.9	0.003	0.002	−0.275	−0.001	− 0.7	0.0
Central_east	−0.016	−0.022	0.244	−0.005	−4.6	0.0	0.022	0.016	0.252	0.004	4.8	2.5
Central_west	−0.046	−0.068	− 0.262	0.018	15.2	8.9	−0.013	−0.009	− 0.265	0.002	2.7	1.4
South_east	−0.052	−0.071	0.035	−0.002	−2.1	0.0	−0.053	− 0.034	−0.024	0.001	0.9	0.5
South_west	−0.054	−0.078	− 0.078	0.006	5.2	3.0	−0.001	−0.001	0.060	0.000	0.0	0.0
Education Level						**13.8**						**14.0**
Illiterate	0.015	0.060	−0.412	−0.025	−21.3	0.0	0.082	0.091	−0.509	−0.047	−54.6	0.0
Primary	0.038	0.125	−0.008	−0.001	− 0.9	0.0	0.071	0.134	−0.220	−0.029	−34.4	0.0
Secondary	0.042	0.066	0.421	0.028	23.7	13.8	0.052	0.068	0.334	0.023	26.6	14.0
University (ref)						0.0						0.0
Occupation						**13.2**						**13.6**
Unemployed	−0.022	−0.106	−0.216	0.023	19.7	11.5	−0.021	−0.045	− 0.202	0.009	10.7	5.7
Workers	−0.028	−0.084	− 0.041	0.003	3.0	1.7	−0.039	−0.077	− 0.167	0.013	15.0	7.9
Medium	−0.014	−0.006	0.683	−0.004	−3.7	0.0	−0.016	− 0.005	0.506	− 0.002	−2.7	0.0
High (Ref)						0.0						0.0
Household income						**10.8**						**19.2**
Lowest quintile	−0.035	−0.068	−0.993	−0.067	−57.7	0.0	−0.022	−0.022	− 0.999	0.022	25.4	13.4
2	−0.021	−0.041	− 0.507	0.021	17.8	10.3	0.004	0.004	−0.498	−0.002	−2.1	0.0
3	−0.006	−0.011	0.001	0.000	0.0	0.0	0.024	0.023	0.005	0.000	0.1	0.1
4	0.001	0.002	0.498	0.001	0.7	0.4	0.019	0.019	0.498	0.009	10.9	5.7
Highest quintile (ref)				0.000		0.0						0.0
C (conindu)	**0.116**						**0.085**					
Residual	**0.055**						**0.042**					

^a^*Coeff* coefficient, *Elas* elasticity, *C* concentration index, *Cont. to C* contribution to concentration index, *%* percentage contribution, *Adj %* Adjusted percentage

## Discussion

The study revealed a high evolution of cardiovascular risk factors among Tunisian adults during the last decade with substantial pro-rich income-related inequalities. Similar upward trends have been previously been shown [30, 31] particularly in low and middle-income countries [32, 33].

We have used the two most common methods to measure and explain health inequalities, the global sensitivity analysis of logistic regression model and the Wagstaff-type decomposition analysis using the cardiovascular risk factors in the last decade as outcomes in Tunisia. To the best of our knowledge, this is the first study comparing these two methods mathematically and epidemiologically in Tunisia and the Maghreb region.

This study highlights that the results provided by the GSA and Wagstaff decomposition analysis methods show that higher risk for cardiovascular diseases is concentrated among those with higher socio-economic status in 2005. Similar results were observed in 2016. Moreover, both methods show similar factors explain the inequalities (income, educational level and regional conditions (area and region)) but with different rankings of importance.

An interesting result from this analysis is that those with high socio-economic status have a higher risk for cardiovascular diseases and diabetes than those from lower socio-economic groups in both 2005 and 2016, except for hypertension. Hypertension was the most prevalent disease in the lowest socio-economic groups in 2016 and the use of tobacco in 2016, was most common in those with income in the mid-range. The findings of the present study are generally in line with another Tunisian study which showed that those with high living standards had a three times higher risk of developing a cardiovascular event in 10 years than those with low living standards [34].

Result are also consistent with the descriptive study conducted in 2011 in Morocco that showed that cardiovascular risk increased proportionally with household income [35].

In contrast to our results, other studies from low and middle-income countries have shown that cardiovascular risk factors are often higher in groups with low socioeconomic status than in those with high socioeconomic status [36–38]. These studies have suggested that observed social gradient depends on the economic and social context of the country.

It is known that income-related inequalities in cardiovascular risk factors and diabetes exist in all countries and are considered unacceptable [39–44]. The challenge facing policymakers is, to ensure that strategies for reducing inequalities must be targeted and justified by rigorous research.

A study in Swedish middle-aged women and men showed that the magnitude of income-related inequalities in CVRFs and their determinants differed between the risk factors and gender. Income was the dominant factor for BMI, abdominal obesity, triglycerides, glucose regulation and LDL-cholesterol, explaining between 30 and 49% of the inequality, whereas education was more important for HDL-cholesterol and total-cholesterol (explaining 24.3 and 41.0% respectively), and occupation was more important for blood pressure (explaining 47.3%) [45].

We found similar results in our study. Income was the major factor for obesity, hypertension hypercholesterolemia and diabetes in Tunisia in 2016, and education was more important for tobacco use.

A key priority message of this study is that the income-related inequalities of cardiovascular risk factors and diabetes are explained by regional disparities, education level and income.

Thus, in Tunisia, promoting equity through universal coverage and improving access to quality care, are two fundamental elements to help remedy the situation and interventions should specially be targeted to high risk groups [46].. It is also important to improve the governance of the system and to make the multisectoral approach a reality for a more effective response to major health problems, such as cardiovascular disease and diabetes.

In term of methodology, several studies in the field have used a simple logistic regression for the study of association between the outcome and determinants Where the results are interpreted in terms of odds ratio (OR) [9, 10, 47].

To overcome the limitations of the single logistic regression some studies develop a global sensitivity analysis to assess the relative importance of input parameters in the system model and the relative importance of variables allowing analysis of the robustness of the logistic model [28, 48].

Unfortunately, this approach also has some limitations as it based on the sampling of the distribution function and is not intended to identify the cause of the input variability. It only indicates the type and extent of impact on the model output. Therefore, it cannot be used to determine the source(s) of variance.

Other advanced and recent methods have also been developed to study income-related inequalities such as Wagstaff’s decomposition analysis [29]. This approach should help to overcome the limitations of the previous approach. This method demonstrates that the health concentration index can be decomposed into the contributions of individual factors to income-related health inequality, in which each contribution is the product of the sensitivity of heath with respect to that factor (the elasticity) and the degree of income-related inequality in that factor (the respective concentration index).

These two methods differ on the mathematical and statistical level; however, they can be complementary in application to public health and help address the expectations of decision-makers.

The GSA and the decomposition gave similar results, however, the decomposition requires much more information to be performed and is mathematically a more complex method.

Therefore, in settings where: a) information is not routinely collected or data do not match the standard requirements to run a decomposition or b) the statistical skills are not in place, then a GSA could be carried out similar results are shown.

## Conclusions

Our study revealed considerable inequalities in cardiovascular risk factors and diabetes in Tunisia in 2005 and 2016, before and after the revolution. The results suggest that most of this inequality can be attributed to socio-economic factors, regional conditions (region and area of residence) and demographics.

The results, therefore, suggest that income and educational level inequalities as well as regional conditions (area and region) represent the main factors responsible for the income-related inequalities in cardiovascular risk factors and diabetes in Tunisia during the last decade.

The results to explain the income-related inequalities of cardiovascular disease and diabetes in Tunisia over time are consistent across the two methods used, the global sensitivity analysis of logistic regression and the decomposition of the concentration index. The findings contribute to gaps in the literature concerning the analysis of social determinants in mathematical and epidemiological terms.

This study argues that, although, the two methods used are mathematically different in nature, their results are complementary and can provide valuable explanations to the observed social inequities of cardiovascular risk factors and diabetes.

Thus, these findings suggest that addressing the root cause of inequality is essential for promoting cardiovascular risk factors and diabetes equity in Tunisia.

The information provided by the analysis might be helpful to identify potential targets for health interventions in order to reduce these income-related inequalities in Tunisia and the methods can be applied to other health problems and populations.

## Data Availability

For TAHINA-2005 survey, the datasets used and analyzed during the current study are available from the corresponding author on reasonable request. For THES-2016 survey, The Tunisian Ministry of Health/National Institute of Health and World Health Organization will publish the datasets online.

## Abbreviations

ADA: American Diabetes Association
BMI: Body Mass Index
C: Concentration index
CC: Concentration Curve
CVDs: Cardiovascular Diseases
DBP: Diastolic Blood Pressure
FPG: Fasting Plasma Glucose
GSA: Global Sensitivity Analysis
HbA1C: Hemoglobin A1C
OR: Odds Ratio
SBP: Systolic Blood Pressure
s_i_: Sensitivity index
TAHINA-2005: Epidemiological Transition and Health Impact in North Africa Survey-2005
THES-2016: Tunisian Health Examination Survey-2016
WHO: World Health Organization
